# Proatherogenic Sialidases and Desialylated Lipoproteins: 35 Years of Research and Current State from Bench to Bedside

**DOI:** 10.3390/biomedicines9060600

**Published:** 2021-05-25

**Authors:** Alexandre Mezentsev, Evgeny Bezsonov, Dmitry Kashirskikh, Mirza S. Baig, Ali H. Eid, Alexander Orekhov

**Affiliations:** 1 The Institute of General Genetics, Moscow 117809, Russia; 2 The Institute of General Pathology and Pathophysiology, Moscow 125315, Russia; evgeny.bezsonov@gmail.com (E.B.); dim.kashirsckih@gmail.com (D.K.); a.h.opexob@gmail.com (A.O.); 3 Department of Biology and General Genetics, I.M. Sechenov First Moscow State Medical University (Sechenov University), 8 Izmailovsky Boulevard, Moscow 105043, Russia; 4 Department of Biosciences and Biomedical Engineering (BSBE), Indian Institute of Technology Indore (IITI), Simrol, Indore 453552, India; msb.iit@iiti.ac.in; 5 Department of Basic Medical Sciences, College of Medicine, QU Health, Qatar University, P.O. Box 2713, Doha 2713, Qatar; ali.eid@qu.edu.qa; 6 Biomedical and Pharmaceutical Research Unit, QU Health, Qatar University, P.O. Box 2713, Doha 2713, Qatar

**Keywords:** atherogenesis, desialylation, LDL, HDL, apolipoproteins, sialidase, trans-sialidase, anti-LDL antibodies, animal models of atherosclerosis, diagnostics of atherosclerosis, treatment options for atherosclerosis

## Abstract

This review summarizes the main achievements in basic and clinical research of atherosclerosis. Focusing on desialylation as the first and the most important reaction of proatherogenic pathological cascade, we speak of how desialylation increases the atherogenic properties of low density lipoproteins and decreases the anti-atherogenic properties of high density lipoproteins. The separate sections of this paper are devoted to immunogenicity of lipoproteins, the enzymes contributing to their desialylation and animal models of atherosclerosis. In addition, we evaluate the available experimental and diagnostic protocols that can be used to develop new therapeutic approaches for atherosclerosis.

## 1. Atherogenicity

Atherogenicity is the ability of atherogenic lipoproteins (LP) circulating in the blood to promote the formation of atherosclerotic plaques [1]. The appearance of foam cells in the arterial wall is probably the earliest known event in the pathogenesis of atherosclerosis and the modified atherogenic low-density lipoprotein (LDL) is the main source of accumulating lipids in these foam cells.

In healthy individuals, the interaction of LDL with the artery cells is mediated by the specific receptor LDLR. It does not lead to an excessive deposition of intracellular lipids. The native LDL is not atherogenic since it does not induce the formation of foam cells. Some lipid components of LDL are utilized by the cells, and the excess of the lipids is removed.

In patients, changes in the composition of LDL due to its preliminary chemical modification facilitate the self-association of LDL causing its enlargement in size. The enlarged LDL stimulates phagocytosis that, in turn, activates the secretion of proinflammatory factors. These proinflammatory factors promote a development of the inflammatory response and stimulate the accumulation of lipids in the artery wall.

## 2. Desialylation of Lipoproteins as a Risk Factor in Atherosclerosis

Although high levels of cholesterol associated with LDL (so-called “bad cholesterol”) is one of well-known risk factors for heart disease, about 50% of initial cardiovascular events occur in people with normal LDL levels [2]. For this reason, it is a common belief that atherosclerosis is caused by alterations in composition of LDL rather than by elevation of plasma LDL. In 1989, we were the first who reported that the plasma of individuals with cardiovascular disorders had a higher atherogenicity compared to the healthy control group [3]. We also found that the treatment of LDL with sialidase significantly increased the deposition of cholesterol to cultured human aortic intimal cells [3] and promoted the transformation of artery cells, primarily, pericytes, endothelial cells and macrophages into foam cells [4,5]. Moreover, we showed that the level of sialic acid in LDL particles of the individuals with coronary artery disease (CAD) was several-fold less compared to the healthy control group. Based on these findings, we hypothesized that desialylation of LDL makes them atherogenic.

## 3. Chemical Modification of Lipoproteins and Its Impact

Sialic acids (Sia/N-acetylneuraminic acid/Neu5Ac) are a group of negatively charged amino sugars. In the extracellular matrix, the plasma and the glycocalyx, they are covalently bonded to lipids and glycoproteins. The terminal residues of Sia act as ligands to the cellular receptors [6]. They also regulate the retention of apolipoproteins in the circulation [7]. Cleaving the terminal Sia changes the charge of the glycan and causes conformational changes. In turn, these conformational changes are capable of preventing the interaction of the glycan with a cellular receptor. They can also modulate the other biological effects of the desialylated molecule such as cell-cell adhesion, inflammation, binding of calcium ions, prevention of proteolytic degradation of glycoproteins etc. For these reasons, desialylation plays an important role in the pathogenesis of many disorders, including atherosclerosis.

The major protein constituents of LDL, namely apolipoprotein B-100 and GM2 gangliosides are sialylated [8,9]. For instance, there is about a dozen of Sia in the core protein of LDL, APOB100 that is covalently bonded to the termini of its N-linked glycans [8]. Their removal changes the chemical and biological properties of APOB100. Particularly, the APOB100-containing lipoproteins (LP), primarily very low density lipoproteins (VLDL) and intermediate density lipoproteins (IDL) become more susceptible to self-association [10,11]. Desialylation of APOB100 also stimulates the accumulation of cholesterol by smooth muscle cells of human aortic intima and increases the uptake of cholesterol by human macrophages in vitro and in vivo [11]. Desialylated (atherogenic) LDL is smaller and its density is higher compared to LDL obtained from healthy individuals. Desialylated LDL contains less neutral carbohydrates and major lipids. It also contains less vitamins and antioxidants. In addition, it is more susceptible to copper-dependent oxidation [12] and self-association [13].

The other modifications that LDL may undergo in vivo include glycol-oxidation [14], glucosylation [15], oxidation [16] and deglycosylation [11] also make it atherogenic. Moreover, glucosylation of desialylated LDL increases its atherogenic potential [13]. For the individuals with diabetes mellitus, the latter suggests glycosylation as an additional risk factor for atherosclerosis [13]. In turn, the decisive role of desialylation in gaining atherogenicity by LDL is proven by the fact that either degalactosylation of already desialylated LDL, i.e., the cleavage of galactose (Gal) that was covalently bonded to the terminal Sia or their complete deglycosylation, i.e., getting rid of carbohydrates, does not increase atherogenicity of LDL [17]. In addition, free sialic acid protects the artery walls from the development of neointima competing with LDL for the binding to fibrinogen [18].

## 4. Immunogenicity of Desialylated Lipoproteins

Although the specific anti-LDL antibodies are present in the plasma of healthy individuals [19], their level significantly increases in the plasma of CAD patients [20]. These antibodies have different specificities and recognize different epitopes. For instance, a chemical modification of purified LDL with malondialdehyde (MDH-LDL) demonstrated that the antibodies were potentially capable of reacting with short fragments of oxidized polyunsaturated fatty acids covalently bonded to apolipoprotein B [21].

The experiments performed in our lab discovered that anti-LDL antibodies had a much higher affinity to desialylated LDL, compared to LDL modified in any other way, including oxidized LDL [20]. Moreover, the anti-LDL antibodies recognized neither HDL nor LDL obtained from healthy individuals. Binding of anti-LDL antibodies to atherogenic LDL transformed the LDL into the low-density lipoprotein—circulating immune complex (LDL-CIC) and increased immunogenicity and atherogenic potential of LDL. Particularly, LDL-CIC promoted the accumulation of cholesterol and deposition of collagens and glycosaminoglycans in cultured vascular endothelial cells [22,23] as well as increased their proliferation rate [20].

Later, the deposits of LDL-CIC were also discovered in vascular atherosclerotic lesions [24,25,26]. In the artery wall, the presence of LDL-CIC stimulated the deposition of the extracellular matrix (ECM) and stimulated the influx of cholesterol to the macrophages. In addition, accumulation of LDL-CIC promoted the conversion of macrophages into foam cells [27,28]. It is believed that anti-LDL antibodies are produced in vivo in response to desialylation of LDL because desialylated LDL exhibits higher immunogenicity and atherogenicity compared to native LDL [29,30]. In turn, even increased atherogenicity and immunogenicity of LDL-CIC, compared to desialylated LDL suggests an existence of a positive feedback between the production of anti-LDL antibodies and the increase of LDL-CIC in the plasma. In other words, more LDL-CIC is present in the circulation than more anti-LDL antibodies are produced. However, the picture would be incomplete if we did not mention low affinity of anti-LDL antibodies to native LDL [31] that does not exhibit yet atherogenic potential. Binding to the epitopes in native (non-atherogenic and non-immunogenic) LDL would convert native LDL particles to LDL-CIC with high immunogenicity and atherogenicity accelerating the immune response and making them involved in the pathogenesis of atherosclerosis. 

Importantly, these theoretical assumptions were confirmed experimentally [28]. First, the authors of the cited paper demonstrated that LDL-CIC were capable of inducing of atherosclerotic activities causing an increase in the intracellular contents of free and esterified cholesterol in normal intimal smooth muscle cells cultured from undamaged areas of human aorta. Second, they found that the selective elimination of LDL-CIC from the circulation dramatically reduced atherogenicity of the remaining LDL. Third, they showed that CIC-LDL differed in many aspects from native LDL. The level of Sia in these particles was low. It was comparable to one in desialylated LDL. The removed CIC-LDL contained fewer neutral lipids and phospholipids. They were of a smaller size and higher on their density. They are also more electronegative and, respectively, they exhibited a higher electrophoretic mobility [28].

The atherogenicity of CIC-LDL can be also modulated by changing the glycosylation pattern of the anti-LDL antibodies. This suggests another layer of complexity in the regulation of their atherogenic potential. The process of protein glycosylation occurs in the endoplasmic reticulum, where a glycan containing N-acetylglucosamine, mannose, and glucose is transferred to the newly synthesized protein [32]. After trimming, the glycosylated protein is transported to the Golgi apparatus. In the Golgi apparatus, the glycans are getting modified by glycosyltransferases that attach to them the residues of fucose (Fuc), Gal and Sia. In turn, the composition and type of glycosylation influence protein functions. Particularly, they affect the protein conformation and its ability to bind to the other proteins. Specifically, the composition of the glycans, the order of carbohydrate residues and the numbers of glycans attached to the protein will determine its efficiency and stability.

Desialylation/sialylation of the Fc domain is a common mechanism that modulates the proinflammatory properties of the antibody. Although desialylation decreases the affinity of the Fc domain to the specific receptors [33], blocking the sialylation of antibodies using the genetic technologies enhances the inflammatory response. At the same time, the sialylated IgG produces the opposite effect in vivo [34]. In humans, sialylation of N-glycans reduces the risk of cardiovascular disorders [35] and the appearance of atherosclerotic lesions in the carotid artery. Moreover, the sialylated antibodies are less immunogenic [36].

The presence or absence of the other specific carbohydrates in the glycans, such as Fuc is also important for the functionality of antibodies. Defucosylation significantly increases the antibody-dependent cellular cytotoxicity (ADCC) and promotes phagocytosis [37]. Moreover, the defucosylation of antibodies improves their interaction with the low affinity Fc specific receptor FCGR3A [38]. In contrast, the fucosylation of antibodies has a negative effect on ADCC and reduces the affinity of the antibodies to the named receptor [39].

Although the glycosylation of antibodies is subjected to a strict control at different levels (reviewed in [40]), the changes in their glycosylation patterns occur in chronic conditions, during the development of the inflammatory response. In some autoimmune disorders, the glycans contain less Gal residues, compared to the control [41]. In contrast, a higher galactosylation of antibodies is associated with a reduction of their inflammatory activity [42,43]. For instance, a reduced representation of Gal and Sia was discovered in the antibodies of individuals with rheumatoid arthritis compared to healthy controls [44].

## 5. Enzymes Implicated in Desialylation

Previous studies performed by us [45] and others [46,47,48] led to the detection of sialidase activity in the plasma. We were the first who purified the enzyme responsible for desialylation of LP and described this protein as plasma “trans-sialidase”. We assessed its molecular weight (96 kDa) and characterized the pH optimum. We also found that its activity was enhanced in the presence of Ca^2+^ [49]. Moreover, the named enzyme exhibited a much higher affinity to LDL, compared to HDL. In LP, the enzyme hydrolyzed α2,6 bond that connected the residues of Sia to the glycoside moiety. At the same time, the proposed human plasma trans-sialidase also hydrolyzed α2,3 and α2,8 bonds, while to a lesser extent. Analyzing the mechanism of the enzyme reaction, we found that the enzyme transferred sialidase residues to the plasma proteins, primarily, fetuin and transferrin. We also found them in gangliosides. In addition, we showed that the interaction of LDL with “trans-sialidase” in vitro induced the accumulation of esterified cholesterol in human aortic intimal smooth muscle cells [45].

**Figure 1:**
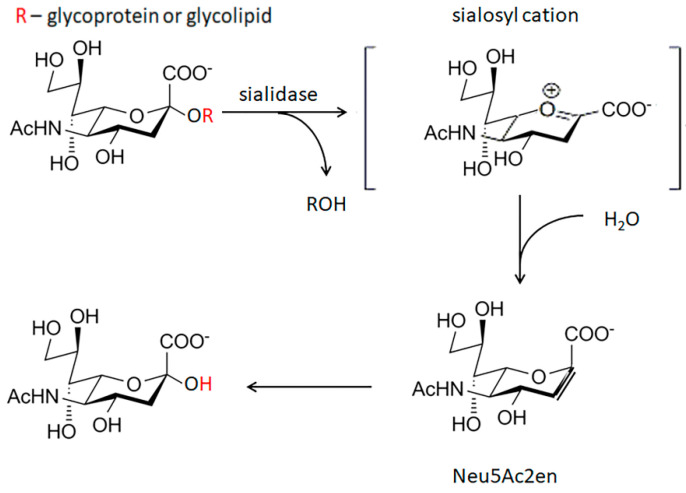
The mechanism of enzyme reaction catalyzed by sialidase. Sialidase hydrolyzes the terminal Sia residue of glycolipids and glycoproteins. Sialosyl cation (in the square brackets), the transition state complex of the reaction; Neu5Ac2en-2-deoxy-2, 3-didehydro-D-N-acetylneuraminic acid, the unsaturated derivative of sialic acid.

Although, the exact identity of the protein responsible for desialylation of LDL still has yet to be revealed, several groups of enzymes that are capable of cleaving or transferring sialidase, namely sialidases (neuraminidases) and trans-sialidases can contribute to this process. Sialidases, which are exoglycosidases, cleave the α-glycosidic linkages of Sia/Neu5Ac (Figure 1). Trans-sialidases, which are mainly present in protozoans, transfer Sia from one sialogalactoside to another via a reverse sialylation of CMP [50]. In addition, viral and bacterial sialidases can be also involved. Alternatively, it can also be either protein(s) with miscellaneous sialidase activity (e.g., KLOTHO [51]) or catalytically active antibodies (abzymes).

From a side, it seems that human sialidases are not involved in desialylation of LP. NEU1, which is also known as lysosomal sialidase, degrades glycoproteins in the lysosomes. To remain catalytically active, NEU1 interacts with cathepsin A, CATHA that protects NEU1 from the degradation [52,53]. NEU2 is constitutively expressed at a low level in the cytoplasm where it recognizes misfolded glycoproteins. NEU3 is located in the cellular membrane [54]. However, it has narrow substrate specificity. NEU4 is found in several intracellular organelles, such as mitochondria and lysosomes. Moreover, three of four human sialidases, namely NEU1, -3 and -4 have acidic pH optimums (4.5–4.8).

On the other hand, NEU1, NEU2 and NEU4 can be recruited to the cellular membrane of various blood cells [53]. For instance, NEU1, CATHA and elastin binding protein (EBP) constitute an elastin receptor complex on the cellular membrane [55] of circulating monocytes and tissue macrophages and can access LP. Moreover, NEU1 and NEU3 are found in exosomes [56]. In this regard, they can contribute to the development of atherosclerosis by removing Sia residues from glycoproteins and glycolipids [57].

## 6. Contribution of Viral Sialidases

**Figure 2:**
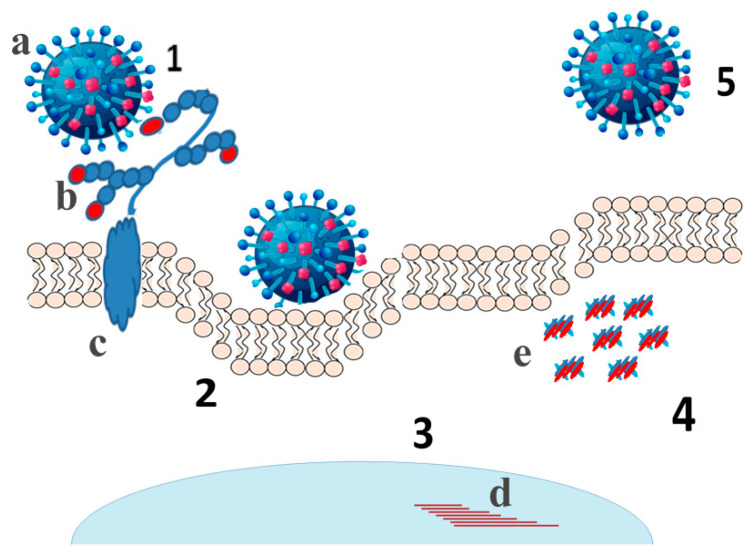
The life cycle of influenza virus. Approaching the cell surface the virus (**a**) cleaves Sia residues (**b**) by viral sialidase from the decoy receptors (1) allowing the viral hemagglutinin to interact with the entry receptors and enter the host cell by endocytosis (2). After releasing from the endosome viral RNAs translocate to the nucleus (3) to replicate the genome and synthesize mRNAs (**d**). Then, viral mRNAs translate to viral proteins in the cytoplasm and viral mRNAs and proteins (**e**) assemble virions (4) that quit the cell by budding. The released virions infect the other host cells (5). (**c)** - transmembrane domain of the decoy receptor.

Sialidase plays an important role in the life-cycle of some viruses [58], such as influenza virus (Figure 2). To clarify whether viral sialidase represents an additional risk factor for cardiovascular diseases, such as atherosclerosis, we evaluated changes in sialidase activity during and after the flu season. We found that in 40–45% volunteers experienced flu symptoms, the activity of viral sialidase increased by factor 2–3. Moreover, their total sialidase activity correlated with viral mRNA encoding sialidase suggesting that, in the human blood, viral sialidase is an additional atherogenic factor [59]. In addition, influenza virus aggravates the apoptosis induced by oxidized LDL in human endothelial cells [60] increasing the probability of blood clog [61].

These results are consistent with other findings. The incidence of admissions due to acute myocardial infarction is 6 times higher among the patients that contracted the flu virus [62] and vaccination against it substantially decreases the risk [63,64,65,66,67]. The flu virus is considered as a risk factor for other cardiovascular events, namely myocarditis, ventricular arrhythmia, and heart failure [68]. Moreover, similar results were obtained for the other viruses [69] and bacteria [70,71,72,73] that express either sialidase or trans-sialidase. Respectively, the chronic infectious diseases that may increase the risk of stroke include periodontitis, chronic bronchitis and infection with Helicobacter pylori [74].

## 7. Abzymes Exhibiting Sialidase Activity

The catalytic antibodies, also known as “abzymes”, were discovered by Tramontano A. et al. in 1986 [75]. Abzymes were found in various autoimmune disorders, such as systemic lupus erythematosus [76], multiple sclerosis [77] and asthma [78]. The catalytic IgG antibodies capable of hydrolysing the terminal Sia of glycoproteins were firstly isolated by Bilyy R. et al. from the blood of the individuals with multiple myeloma [79] and systemic lupus erythematosus [80]. The catalytic mechanism of the abzyme is similar to that of human sialidases since the reaction was inhibited by pan-sialidase inhibitor 2,3-dehydro-2-deoxy-N-acetylneuraminic acid (DANA) and the pH optimum of the abzyme (4.5–6.0) was close to the pH-optimums of human sialidases. In vitro, these antibodies were capable of desialylating glycolipids and glycoproteins located on the surface of human red blood cells and in vivo, they also activated phagocytosis [80].

## 8. Multiple Modification of LDL in the Blood

Chemical modification of LDL is a cascade of well-arranged changes that includes desialylation, partial loss of the lipids, reduction of the particle size, acquiring a negative charge and oxidation, and peroxidation of the oxidized lipids. Although desialylation of LDL is the initial step of their modification, loss of Sia makes LDL atherogenic. The following steps also increase its atherogenicity [22]. The other non-enzymatic and enzymatic modifications can contribute to the modification of LDL and promote the accumulation of lipids by artery cells. Moreover, the modification of LDL does not stop after attachment of the particle to the arterial walls [81].

The existence of multiple steps explains the heterogeneity of modified LDL in the blood of the individuals with atherosclerosis and/or diabetes. Following the described order, the modification of LDL particles facilitates their self-association. Concurrently, cholesterol and cholesterol esters conjugate with apolipoproteins. Since their accumulation increases, it causes conformational changes in apolipoproteins increasing the immunogenicity of LP and making them a target for autoantibodies [22].

## 9. Changes in Glycosylation of HDL

Making desired changes in the composition of LP, such as reduction of LDL and increase of HDL, is often considered as a beneficial therapeutic approach for cardiovascular diseases. HDL plays a crucial role in the reverse transport of cholesterol and cholesterol esters transferring them from the peripheral tissues to the liver. HDL also reduces the deposition of cholesterol in the arterial wall preventing the transformation of artery cells into foam cells [82]. Moreover, HDL possesses anti-inflammatory [83,84], antioxidant [85], antimicrobial [86] and vasodilating [87] activities.

The plasma HDL is heterogeneous. There are several subclasses of HDL with different biological activities. Moreover, they undergo significant remodeling in vivo. As with other LP, the HDL particles have different size, charge and density. Their composition is also different (reviewed in [88]). According to the references, a small, dense HDL that is enriched in proteins, known also as HDL3c, protects LDL from oxidation. Transferring the oxidized lipids from LDL to HDL is the first step of HDL-mediated protection from oxidative damage. Inactivation of oxidized lipids by HDL represents the second step in this protective pathway [89,90]. The levels of aldehydes and oxidized short-chain phospholipids in HDL decreases due to subsequent inactivation by reduction to inactive hydroxides [90]. In addition, HDL3 acquires oxidized lipids from the cellular membrane [90].

Increasing atherogenicity of LDL, desialylation is also making HDL less anti-atherogenic [17]. Primarily, desialylation of native HDL diminished its capacity to efflux cellular cholesterol from artery cells [17]. For instance, the macrophages possess two mechanisms to efflux cholesterol. First, the transfer of cholesterol from the cell can be mediated by the scavenger receptor SR-BI due to the gradient of concentration. Second, cholesterol can be transferred from the cell by the transporter ABCA1 in the ATP-dependent manner [91,92]. Since desialylation of HDL impairs both mechanisms [17], the transfer of cholesterol through the receptors relies on sialylation of HDL [93].

Moreover, desialylation of apolipoprotein E (ApoE), a protein component of HDL, inhibits its incorporation into HDL impairing the reverse cholesterol transport [94]. It also alters the interaction of apolipoprotein A1 (ApoAI) with cellular proteins, including ABCA1 and inhibits the association of HDL with lipases [94]. In addition, desialylation of HDL inhibits heavily N-glycosylated lecithin–cholesterol acyltransferase (LCAT) that mediates esterification of cholesterol [17,95].

### 9.1. Coronary Artery Disease

HDL particles obtained from the individuals with cardiovascular disorders are compositionally different from healthy control. They contain less apolipoproteins, including ApoAI, ApoAII, and ApoE. They are also enriched in C3, a protein essential for the activation of complement pathways (classical, alternative and lectin pathways) [96]. Moreover, the patients’ HDL contains oxidized ApoAI and higher levels of ApoCIII.

The changes observed in the patients’ HDL alter their functionality [97]. For instance, HDL proteome profile is enriched by proinflammatory proteins [96], such as the acute phase response protein SAA2 that inhibits the efflux of cholesterol to HDL and decreases the ability of HDL to remove cholesterol from the artery cells [98]. Moreover, low levels of ApoAI and its oxidation are considered as risk factors for the patients, since they diminish cardioprotective, and antiatherogenic effects of HDL [99].

### 9.2. Type II Diabetes

The composition and biological functions of HDL are under a tight genetic control [100,101,102,103,104]. The biological effects of small, dense HDL are changed markedly in the individuals with insulin resistance and chronic inflammation [105,106,107]. Patients with type II diabetes typically have dyslipidemia characterized by high triglycerols and low HDLC levels [106]. Their altered glycemic status preferentially affects small, dense HDL3c particles. These particles display distinct compositional alterations due to an increased activity of cholesteryl ester transfer protein (CETP) [85]. In the patients with poor glycemic control, these changes coincide with reduced ability of HDL3c to prevent oxidation of LDL [106]. Moreover, the replacement of ceramides by triglycerides in their lipid core results in a reduced penetration of ApoAI into the lipid phase [108], impairing the functionality of HDL [106,109,110]. In addition, the enrichment of HDL3 in triglycerides is accompanied by the loss of ApoAI by these particles [85].

### 9.3. Familial Apolipoprotein A-I (ApoAI) Deficiency

Familial apolipoprotein AI deficiency (FAID) is characterized by low levels of both ApoAI and HDL cholesterol and is associated with accelerated atherosclerosis. ApoAI is the major protein of HDL, comprising ~35% of total HDL mass and ~70% of HDL protein [111]. Respectively, ApoAI deficiency in the patients’ blood impairs HDL structure, composition, metabolism and function.

The previous studies discovered that HDL of the patients with nonsense mutation in *APOA1*, (Q(-2X) contained less ApoAI, phospholipids and cholesteryl esters and more ApoAII, free cholesterol and triglycerides compared to healthy controls. The altered lipid and apolipoprotein composition correlated with a deficiency of intrinsic atheroprotective properties, such as reduced antiatherogenic activity as HDL as well as small, dense HDL3 [112,113].

In continuation of these studies, we found that the most prominent alterations were observed in phospholipids and sphingolipids species possessing multiple unsaturations in their fatty acid residues [97]. We confirmed that the alterations in HDL content reduced anti-atherosclerotic activities of HDL. In this regard, the abundances of the species that were decreased in ApoAI–deficient HDL discovered positive correlations with the HDL functional metrics, whereas negative correlations were observed for the species whose HDL content was increased. For instance, the increased amounts of proinflammatory lipids, such as lysophosphatidylcholine and phosphatidic acid, made ApoAI–deficient HDL more susceptible to oxidation. In contrast, the altered biological functions of HDL in ApoAI–deficient individuals did not include the ability to suppress apoptosis because ApoAI–deficient HDLs contained the normal level of S1P per a protein unit.

To summarize the importance of desialylation for functioning of the arteries, we would like to raise a few important questions that urgently require to be answered. First, we would know whether the modification of LP is reversible. The ability to delay it or turn it over would revolutionize the treatment of cardiovascular diseases substantially prolonging human lives. Second, the factors causing desialylation should be uncovered, their role has to be revealed and their potential harm needs to be assessed. The knowledge of reasons that cause atherogenicity would allow us to intervene and offer reasonable treatment options to control the disease. Third, we would address the problem of immunogenicity and it would help us to manipulate the production and properties of the specific antibodies in vivo. Answering these questions requires experimental models of higher complexity, such as genetically modified lab animals.

## 10. Animal Models of Desialylation

The previously developed *Apoe*^(-/-)^ and *Ldlr*^(-/-)^ mice with genetic deficiency of *Neu1*, -*3* and -*4* or those treated with specific inhibitors of sialidases often serve us as common models of atherosclerosis [57,114]. *Apoe*^(-/-)^ mice have significantly higher levels of total cholesterol and LDL cholesterol in blood when fed with a high cholesterol diet [114]. *Ldlr*^(-/-)^ mice do not develop spontaneous lesions when fed with a normal rodent diet. However, they develop atherosclerotic plaques similar to those in *Apoe*^(-/-)^ mice, when their food is supplemented with moderate amounts of cholesterol [114,115].

The recent study discovered a new Neu1-dependent pathway that contributes to atherosclerosis [57]. It was shown that an injection of *Apoe*^(-/-)^ mice with desialylated LDL labeled with a fluorescent dye led to an accumulation of LDL in the artery roots and the uptake of LDL by the artery cells was mediated by the lectin receptor Asgr1. The importance of the identified pathway in atherosclerosis was confirmed by other findings including the experiments performed in our lab (e.g., [116]).

To prove the role of Neu1 in atherosclerosis, the progression of atherosclerosis was compared in two species of knockout mice, namely *Neu3*^(-/-)^ *Apoe*^(-/-)^ and *Neu4*^(-/-)^ *Apoe*^(-/-)^ and *CathA*^S190A-Neo^ *Apoe*^(-/-)^ mice, which were deficient in Neu1 by 90%. It was found that either 90% reduction in Neu1 activity or complete inactivation of Neu3 in *Apoe*^(-/-)^ animals slowed down the progression of atherosclerosis. In contrast, the progression of atherosclerosis in *Neu4*^(-/-)^ *Apoe*^(-/-)^ mice was not significantly different, compared to *Apoe*^(-/-)^ animals with normal Neu4 activity [57]. Moreover, the arteries of *CathA*^S190A-Neo^ *Apoe*^(-/-)^ mice were less susceptible to infiltration by macrophages compared to their *Neu3*^(-/-)^ *Apoe*^(-/-)^ and *Neu4*^(-/-)^ *Apoe*^(-/-)^ counterparts suggesting that two different mechanisms should be responsible for the initiation of the inflammatory response and triggering atherosclerosis [57,117]. Particularly, both Neu1 and Neu3 were capable of desialylating LDL whereas Neu1 was also involved in activation of the inflammatory response. 

Although the analysis of mouse plasma did not reveal significant differences in the levels of total cholesterol, LDL cholesterol, HDL-cholesterol or triglycerides between *Apoe*^(-/-)^, and *CathA*S190A-Neo *Apoe*^(-/-)^ mice, a higher sialylation of ApoB was discovered in the plasma of *CathA*^S190A-Neo^ *Apoe*^(-/-)^ mice [57]. This finding indicated that Neu1-deficiency rather than changes in the levels of plasma cholesterol was responsible for a delayed development of atherosclerosis in *CathA*^S190A-Neo^ *Apoe*^(-/-)^ animals. This conclusion was confirmed by the analysis of constitutive *Neu1* knockout mice, namely *Neu1*^ENSMUSE141558^ and *Neu1*^ΔEx3^.

The comparative analysis of the plasma samples obtained from *Neu1* knockout mice, *CathA*^S190A-Neo^ and wild type animals demonstrated a 3-fold increase of LDL in the plasma of *Neu1* knockout mice, compared to the other phenotypes. On the other hand, the reduction of the residual Neu1 activity in the *CathA*^S190A-Neo^ *Apoe*^(-/-)^ mice to 10–20% significantly delayed the atherogenesis without interfering with the LDL level. These results confirmed that Neu1 drives the uptake of LDL and helped to assess the threshold for the inhibition of Neu1 in circulation [57].

However, White with coauthors [118] using hypomorphic NEU1 expression in *Apoe^(^*^-/-)^ mice showed the reduced serum levels of VLDL and LDL cholesterol in these mice. The difference with the study performed by Demina with coauthors [57] was in using 7 months-old mice males [118] instead of less than 4 months-old females [57], analysis of serum [118] instead of plasma [57], and different constructs for hypomorphic NEU1 expression.

**Figure 3:**
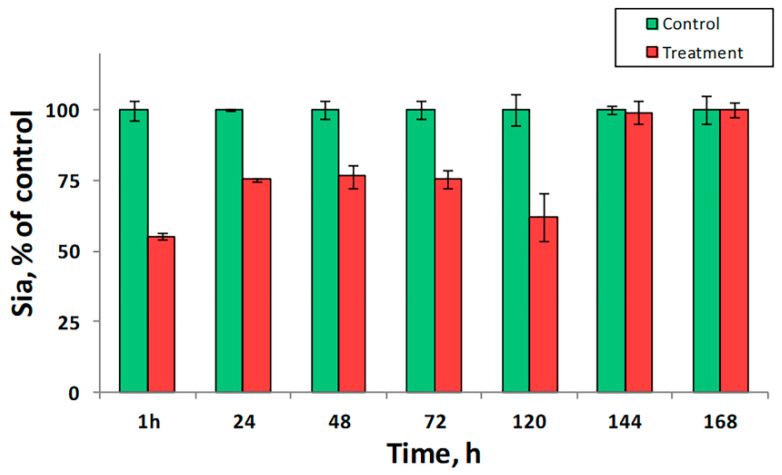
Desialylation of plasma proteins by immobilized sialidase in vivo. Green bars—control animals treated with saline (N = 10); Red bars—animals treated with immobilized sialidase (N = 10). The data are represented as mean ± SE.

To prove the role of sialidase as a proatherogenic factor in vivo, we analyzed the sialylation of LDL after an injection of healthy mice with immobilized sialidase. We found that even a single dose of the preparation reduced the level of Sia in LDL by 50% (Figure 3). The sialylation reached the minimum within an hour suggesting that sialidase activity in the murine plasma was very low and a higher dose of the preparation could be used to treat the animals. We also discovered that the observed effect persisted for five days. In turn, this finding indicated that the replacement/sialylation of LDL was a much slower compared to desialylation. Respectively, we assumed that the capability of blood cells and exosomes to compensate desialylation was insignificant and a pharmacological intervention could be needed to compensate for an increased sialidase activity in vivo.

In turn, the selective inhibition of Neu1 and Neu3 *Ldlr*^(-/-)^ mice fed with a high fat diet and *Apoe*^(-/-)^ mice showed that daily treatment of mice for several weeks decreased Neu1 activity by 70–80% and reduced the size of atherosclerotic lesions by 25–30% [57]. In contrast, the inhibitors did not affect the levels of total cholesterol, LDL cholesterol, HDL-cholesterol or triglycerides in plasma. In addition to that, Bocquet with coauthors [119] discovered that the administration of the sialidase inhibitor oseltamivir phosphate by *Ldlr^(^*^-/-)^ mice fed with high fat diet significantly decreased plasma levels of LDL-cholesterol in these mice. Demina with coauthors [57] did not study effects of oseltamivir phosphate in *Ldlr^(^*^-/-)^ mice and they used another sialidase inhibitors. The existing differences in the literature point in the direction that more research should be done in this field in order to clarify effects of hypomorphic NEU1 expression and sialidase inhibitors on LDL cholesterol levels in mice.

Summarizing the obtained results we would like to highlight the atherogenic and proinflammatory potential of the murine Neu1. The 90% Neu1-deficiency in *CathA*^S190A-Neo^ *Apoe*^(-/-)^ mice delayed the growth of atherosclerotic plaques and the infiltration of the arterial intima by immune cells. We would also mention a preventive effect on atheroma formation of selective Neu1 inhibitors. In the future studies, we aim to prove that Neu1 activity in plasma negatively correlates with atherogenicity. This can be achieved by the maintenance of the enzymatic activity at a certain level using different doses of the specific inhibitors. Alternatively, one of the sialidases can be immobilized on an inert carrier and periodically injected to the bloodstream.

## 11. Clinical Impact of Desialylation

One of the most widely used treatment options for atherosclerosis is lowering LDL levels with statins, which are inhibitors of hydroxymethyl glutaryl coenzyme A reductase. However, statins only benefit only 35% of patients with CAD. Moreover, >20% of patients experience a recurrent event within 2–3 years of an acute coronary syndrome, despite receiving high-doses of statins [120]. Together, the evidence presented and discussed above underline the necessity of finding new therapeutic targets for atherosclerosis.

Chemical modifications of LP can be used to diagnose cardiovascular disorders, evaluate the available treatment options and treat the diseases. To date, several methods were developed to screen panels of experimental drugs for the compounds with antiatherogenic activity [121,122,123]. Some other techniques allow to assess trans-sialidase and sialidase activities in the plasma [124], to quantify modified LDL [125,126,127], LDL-CIC [128,129,130] and anti-LDL antibodies [23,131].

The data obtained by us and the others suggest that new therapeutic approaches may involve a transfusion of lipoprotein particles containing anti-atherogenic plasma lipids, apolipoproteins and plasma enzymes. To date there are several examples of successful antiatherogenic therapy with LCAT [132,133] and HDL enriched by native ApoAI [134]. Moreover, lipoprotein particles designated for transfusion can be enriched by vitamins, antioxidants and antiatherogenic lipids, such as the isomer of phosphatidylcholine 34:2, palmitoyllinoleoyl phosphatidylcholine [97]. Moreover, non-lipid atherogenicity factors, such as anti-LDL antibodies can be removed from the patients’ blood using LDL-apheresis [135] and immobilized LDL [135,136]. Alternatively, it may be one of the specific sialidase inhibitors that increases sialylation of LP, reduces the lipid uptake by the artery cells and increases the cholesterol efflux to HDL [137].

## 12. Conclusions

In conclusion, we would like to mention that atherogenicity of plasma LP is probably one of the major vulnerabilities in the human body. In individuals predisposed to atherosclerosis, the LP, primarily LDL and HDL are subjected to multiple chemical modifications and physical transformations that favor their accumulation by foam cells and interfere with the normal flow of their catabolism. A failure to translate the pharmaceutical manipulations with LDL and HDLC levels in plasma into a cardiovascular benefit to the patient requires us to zoom in and take into account the new compelling evidences for the critical role of the enzymes metabolizing lipid and non-lipid constituents of LP in atherogenesis.

In this review, we provide multiple evidences that desialylation of LDL causes the uncontrolled accumulation of lipids by the artery cells. In turn, desialylation of HDL impairs its capability to extract lipids from the peripheral tissues and deliver them to the liver. As we also show, either genetic inactivation of sialidases or their pharmacological inhibition significantly delays the progression of the disease. For these reasons, the future pharmacological research should be extended to regulation of the enzymes involved in the metabolism of glycolipids and glycoproteins rather than remaining focused on stabilization of plasma LDL and HDLC levels.
